# High Prevalence of MRI Features of Mesenteric Panniculitis in Chronic Intestinal Inflammation: A Retrospective 3-T MRI Study

**DOI:** 10.3390/diagnostics16111733

**Published:** 2026-06-04

**Authors:** Vahidreza Tehranirad, Julian Ramin Andresen, Marc Olaf Liedke, Christoph Kopetsch, Fabian Scheer, Reimer Andresen

**Affiliations:** 1 Institute for Diagnostic and Interventional Radiology/Neuroradiology, Westkuestenklinikum Heide, Academic Teaching Hospital of the Universities of Kiel, Luebeck and Hamburg, Esmarchstraße 50, 25746 Heide, Germany; christoph.kopetsch@wkk.sh (C.K.); fabian.scheer@wkk.sh (F.S.);; 2 Department of Orthopaedics, University Clinic for Orthopaedics and Trauma Surgery, Medical University of Vienna, Währinger Gürtel 18–20, 1090 Vienna, Austria; ramin.andresen@meduniwien.ac.at; 3 Department of Visceral, Thoracic and Vascular Surgery, Westkuestenklinikum Heide, Academic Teaching Hospital of the Universities of Kiel, Luebeck and Hamburg, Esmarchstraße 50, 25746 Heide, Germany; marc.liedke@wkk.sh

**Keywords:** mesenteric panniculitis, magnetic resonance imaging, 3-T MRI, inflammatory bowel disease, Crohn’s disease, sigmoid diverticulitis, ulcerative colitis

## Abstract

**Background/Objectives:** Mesenteric panniculitis (MP) is a chronic inflammatory and sclerosing disorder of the mesenteric fat. This study evaluated the occurrence and MRI appearance of MP in patients with chronic inflammatory bowel disease and chronic inflammatory bowel conditions. **Methods:** In this retrospective single-center study, 312 individuals underwent standardized 3-T MRI Sellink examinations, including 252 patients with clinically confirmed chronic inflammatory bowel disease or chronic inflammatory bowel conditions and 60 control patients without intestinal inflammation. MP was diagnosed when at least three of five characteristic MRI findings were present: increased mesenteric signal intensity on fat-saturated fluid-sensitive T2 sequences, fat ring sign, pseudocapsule, embedded micronodules, and displacement of bowel loops. Group differences were analyzed using contingency table analysis with Monte Carlo exact testing; Pearson’s chi-square test and Holm-adjusted pairwise post hoc comparisons were additionally performed. A *p*-value < 0.05 was considered statistically significant. **Results:** MRI signs of MP were present in 221/312 patients (70.8%) overall, including 220/252 patients (87.3%) in the disease cohort and 1/60 patients (1.7%) in the control group. Among patients with MP, the underlying diseases/conditions were Crohn’s disease (104/220, 47.3%), sigmoid diverticulitis (88/220, 40.0%), and ulcerative colitis (28/220, 12.7%). The overall distribution of MP extent differed significantly among diagnostic groups (Monte Carlo exact test: *p* = 0.030), although adjusted pairwise comparisons were not statistically significant. Topographically, MP showed a clear predominance in the left upper quadrant. **Conclusions:** MRI-defined features consistent with mesenteric panniculitis are highly prevalent in this selected cohort of patients with chronic intestinal inflammatory conditions when standardized criteria are applied. These findings suggest that MP-like mesenteric changes may represent a common imaging correlate of chronic intestinal inflammation rather than a rare incidental finding. MRI enables consistent detection and topographic assessment of MP.

## 1. Introduction

Mesenteric panniculitis (MP) belongs to the spectrum of sclerosing mesenteritis, which has been discussed in relation to IgG4-associated fibroinflammatory disorders in parts of the literature [1,2]. Histopathologically, MP is characterized by inflammatory cell infiltration, fat necrosis, and fibrosis of the mesenteric adipose tissue, with these three components occurring to varying degrees [3,4,5,6]. In approximately 90% of cases, the root of the jejunal small-bowel mesentery represents the predominant site of involvement, whereas the mesocolon and mesoappendix are affected less frequently [7,8]. Reported associated conditions include inflammatory, autoimmune, postoperative, and malignant processes [3,7,8,9,10,11,12,13,14,15,16]. In addition, MP has been reported to be associated with breast cancer and multiple myeloma in women and with lymphoma in men [16]. Recent clinical and radiological case series have further emphasized the heterogeneous presentation of MP [17,18], including atypical abdominal pain [19], persistent diagnostic uncertainty between benign inflammatory disease and malignancy [20], and FDG PET/CT appearances mimicking metastatic disease in a pediatric patient receiving immunotherapy [21].

The reported mean prevalence of MP is approximately 1%, with published ranges from 0.16% to 3.3% [6,8,13,22,23,24,25,26]. In a cohort of 5595 patients, mesenteric panniculitis was identified on computed tomography (CT) in 2.55% over a 3-year period [13]. MP may occur in all age groups but is seen predominantly in the sixth to seventh decade of life, with a reported male predominance of approximately 2:1 [2,8,25,27]. The differential diagnosis is broad and includes inflammatory, infectious, neoplastic, vascular–ischemic, and idiopathic disorders [28,29,30].

In its early stages, MP is often clinically silent. With increasing inflammatory remodeling, patients may develop nonspecific abdominal symptoms, including abdominal pain, nausea, vomiting, constipation, and diarrhea. Laboratory abnormalities may include elevated C-reactive protein levels and leukocytosis. With the widespread use of cross-sectional imaging, particularly CT and magnetic resonance imaging (MRI), MP is increasingly detected incidentally, including in asymptomatic patients [7,13,28,29,30]. However, most radiological descriptions of MP are still based on CT rather than MRI.

On MRI, MP can be assessed using criteria analogous to those established in CT. Typically, it appears as a solid mesenteric mass, preferentially located along the mesenteric vessels, with intermediate to low signal intensity on T1-weighted images and high signal intensity on T2-weighted images [7,31]. Areas of very low signal intensity on both T1- and T2-weighted sequences are attributable to fibrotic tissue components, which contain predominantly immobile protons with short T2 relaxation times [7]. In advanced retractile stages, the mesenteric mass may show a stellate configuration with radiating extensions and scattered punctate calcifications [7]. A fibrous pseudocapsule and the fat ring sign can be demonstrated both on CT and MRI and are helpful imaging features for distinguishing MP from malignant mesenteric processes [2,7,22,32]. In addition, small nodules diffusely distributed throughout the affected mesentery may be present. In more advanced cases, mass effect with compression and displacement of adjacent small-bowel loops may occur, which can be well depicted on MRI. Owing to its high soft-tissue contrast, MRI also allows assessment of mesenteric vessels, which are often encased but usually not compressed by mesenteric masses; vascular complications may be suggested by absent flow-related signal on gradient-echo or fat-suppressed T1-weighted sequences [7].

Despite these established imaging features, the MRI phenotype of mesenteric panniculitis in the setting of chronic intestinal inflammatory disease has not been systematically characterized. The aim of the present study was therefore to evaluate the prevalence, imaging phenotype, and topographic distribution of mesenteric panniculitis in patients with clinically confirmed chronic inflammatory bowel disease and chronic inflammatory bowel conditions using standardized 3-T MRI criteria.

## 2. Materials and Methods

### 2.1. Study Design and Ethics

This study was designed as a retrospective single-center analysis. Ethical approval was obtained from the Ethics Committee of the Medical Faculty of Christian-Albrechts-University Kiel (approval code: D 527/22).

The primary endpoint was to assess the prevalence and MRI-based imaging characteristics of mesenteric panniculitis (MP) in patients with chronic inflammatory bowel disease or chronic inflammatory bowel conditions. Patients with a history of abdominal surgery or concomitant malignancy were excluded from the analysis.

### 2.2. Study Population

A total of 312 individuals were included in the study. Of these, 252 patients had clinically confirmed chronic inflammatory bowel disease or chronic inflammatory bowel conditions (Table 1), whereas 60 patients without evidence of intestinal inflammation served as a control group (Table 2). The control group consisted of patients who underwent MRI Sellink examination for clinical indications but showed no clinical or imaging evidence of intestinal inflammation. Clinical indications in the control group included assessment or exclusion of inflammatory bowel disease, suspected small-bowel tumor, gastrointestinal bleeding, and unexplained abdominal pain. The control group was selected on the basis of absent intestinal inflammation and was not formally matched to the disease cohort for age, sex, or BMI. The disease cohort comprised patients with Crohn’s disease (110/252), recurrent sigmoid diverticulitis (99/252), and ulcerative colitis (43/252). The mean age of the disease cohort was 44.3 years (range, 18–77 years).

Age differed significantly between the diagnostic groups (Kruskal–Wallis test, *p* < 0.001), with patients with sigmoid diverticulitis being markedly older than those with Crohn’s disease and ulcerative colitis. The sex distribution of the disease cohort was 146/252 women (57.9%) and 106/252 men (42.1%). Additional clinical characteristics are summarized in Table 3.

According to the German S3 guideline for diverticular disease [33], 78/99 patients (77.8%) with recurrent sigmoid diverticulitis were classified as CDD type 3b and 21/99 patients (21.2%) as CDD type 3c. In order to focus on chronic bowel disease, only patients with recurrent episodes were included, whereas first presentations were excluded. MRI Sellink examinations in this subgroup were not performed as primary diagnostic examinations for first-time acute uncomplicated sigmoid diverticulitis. Rather, MRI was obtained during follow-up, typically in patients with symptomatic recurrence and a prolonged clinical course, with an average disease history of more than three years. CT examinations were available as part of routine clinical care in a subset of these patients, but CT was not a predefined study modality in this retrospective MRI-based analysis.

### 2.3. MRI Protocol

All examinations were performed as standardized MRI Sellink studies on a 3.0 T system (Achieva 3.0 T, Philips Healthcare, Hamburg, Germany). The protocol included coronal T2-SPAIR, coronal FFE, axial diffusion-weighted imaging, and axial T2-TSE sequences. After intravenous administration of butylscopolamine and gadolinium-DTPA, a coronal dynamic THRIVE sequence and axial contrast-enhanced T1-FFE-SPAIR sequences were acquired.

### 2.4. Image Analysis

MRI datasets were pseudonymized and reviewed in a blinded fashion using identification numbers. Image analysis was performed retrospectively by a board-certified radiologist. A renewed pseudonymized re-evaluation of the datasets was additionally performed to assess the robustness of the MRI classification; this reassessment did not materially change the MP classification. MRI findings were considered consistent with MRI-defined MP when at least three of the following five predefined MRI criteria were present:

1. Increased mesenteric signal intensity on fat-saturated fluid-sensitive T2 sequences in the early stage;
2. A pseudocapsule with low signal intensity on T1- and T2-weighted images;
3. Displacement of adjacent bowel loops;
4. Embedded nodules;
5. A fat ring sign around mesenteric vessels and nodules.

Because increased T2 signal intensity on fat-suppressed sequences and displacement of adjacent bowel loops are nonspecific signs, these findings were not interpreted in isolation. Instead, diagnosis required a composite pattern of at least three MRI criteria, with particular attention to more specific morphological features such as a pseudocapsule, embedded nodules, and the fat ring sign.

**Figure 1:**
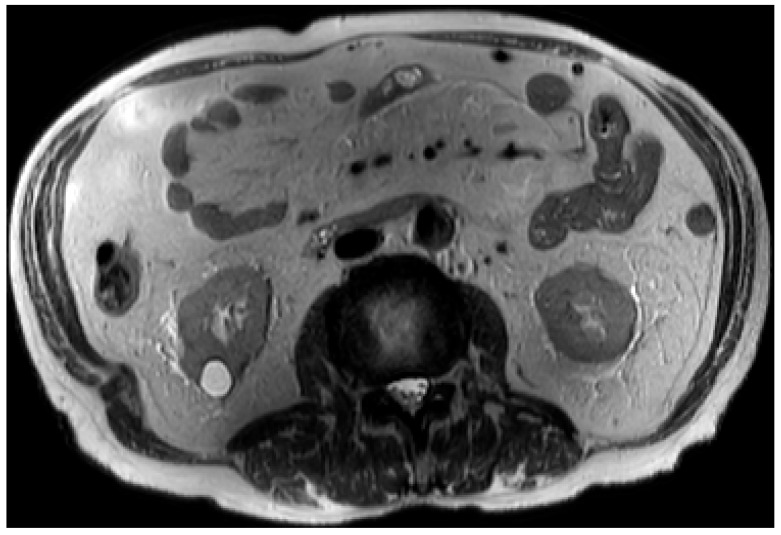
Axial T2-weighted MRI scan demonstrating a low-signal-intensity pseudocapsule, displacement of adjacent bowel loops, embedded nodules, a fat ring sign around mesenteric vessels and nodules, and reduced mesenteric signal intensity, consistent with MP.

An example showing all five MRI features of mesenteric panniculitis is presented in Figure 1.

**Figure 2:**
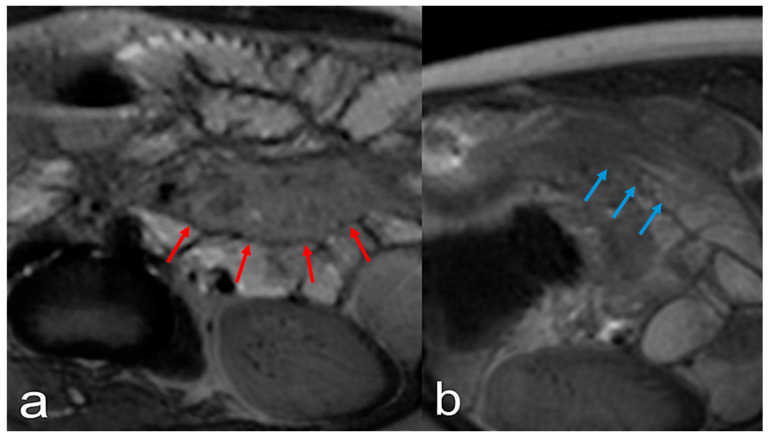
MRI findings in Crohn’s disease: (**a**) axial MRI showing features of MP, including a pseudocapsule (red arrows), central signal reduction in the surrounding fatty tissue, embedded nodules with a halo sign, and displacement of adjacent bowel loops; (**b**) axial MRI showing terminal ileitis with marked bowel wall thickening (blue arrows).

**Figure 3:**
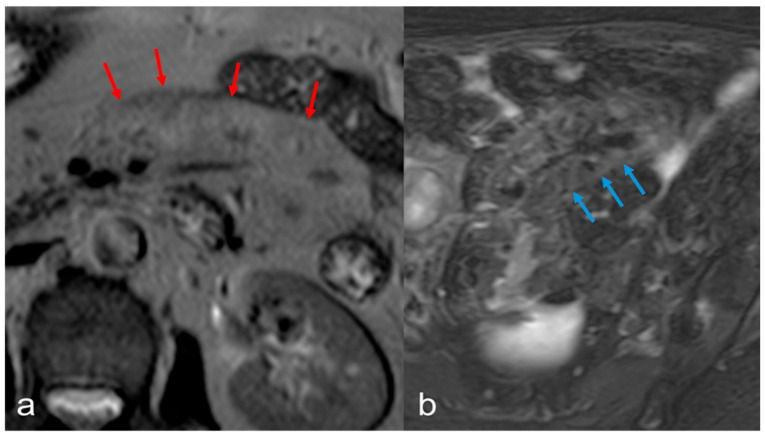
MRI findings in sigmoid diverticulitis: (**a**) axial MRI showing mesenteric panniculitis with a distinct pseudocapsule and mass effect (red arrows); (**b**) coronal MRI showing sigmoid diverticulitis with inflammatory bowel wall thickening (blue arrows).

**Figure 4:**
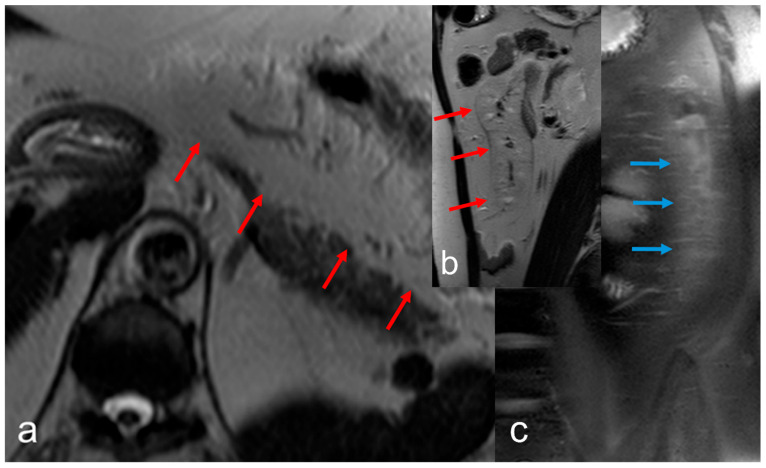
MRI findings in ulcerative colitis: (**a**) axial and (**b**) sagittal MRI showing MRI-defined MP with a pseudocapsule, embedded nodules/fat-ring configuration, and associated bowel-loop displacement/mass effect (red arrows); (**c**) coronal MRI showing inflammatory wall thickening (blue arrows) of the descending colon.

Representative cases of Crohn’s disease, sigmoid diverticulitis, and ulcerative colitis are shown in Figure 2, Figure 3 and Figure 4.

In addition, MP localization and frequency were assigned to abdominal quadrants.

### 2.5. Statistical Analysis

Statistical analyses were performed using SPSS version 23.0 (IBM Corp., Armonk, NY, USA). Categorical variables are presented as absolute and relative frequencies. Differences in the distribution of MP extent (3P, 4P, and 5P) among Crohn’s disease, ulcerative colitis, and recurrent sigmoid diverticulitis were analyzed using contingency table analysis. Because of partially low expected cell counts, Monte Carlo exact testing was used as the primary inferential method; Pearson’s chi-square test was additionally reported. Effect size was quantified using Cramér’s V. Pairwise post hoc comparisons were performed using 2 × 3 contingency tables with Holm correction for multiple testing. All tests were two-sided, and *p*-values < 0.05 were considered statistically significant.

## 3. Results

### 3.1. Manifestation of Mesenteric Panniculitis

Among the 60 control patients without evidence of chronic inflammatory bowel disease, 1 patient (1.7%) fulfilled the predefined MRI criteria for MP, whereas 59/60 patients showed no relevant MRI features suggestive of MP.

**Figure 5:**
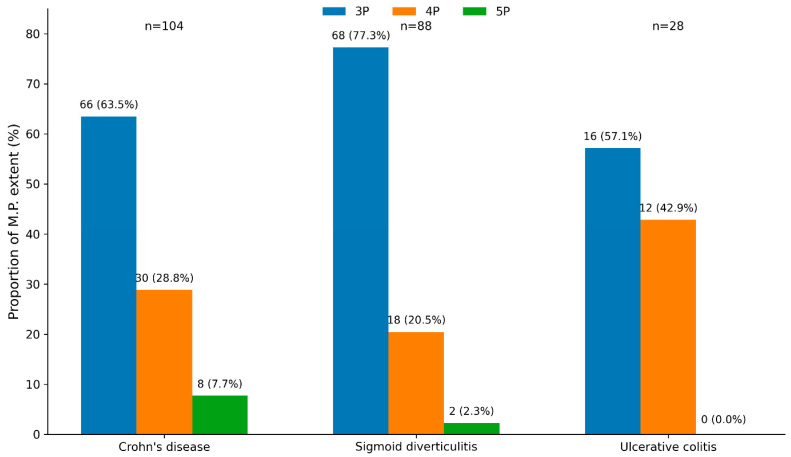
Grouped bar chart showing the proportion of mesenteric panniculitis (M.P.) extent across diagnostic groups. Bars represent the percentage of observations classified as 3P, 4P, or 5P within each disease category. Labels above bars indicate absolute counts and percentages. Total sample size for each diagnostic group is shown above the respective category. No 5P cases were observed in ulcerative colitis. Overall group differences were statistically significant (Monte Carlo *p* = 0.030), with a small effect size (Cramér’s V = 0.155).

In the disease cohort, 220/252 patients (87.3%) fulfilled the predefined MRI criteria for MP. Of these, 150/220 patients had 3 MP criteria, 60/220 had 4 MP criteria, and 10/220 had all 5 MP criteria. Figure 5 illustrates the distribution of MP extent across the diagnostic groups. In all groups, 3P was the most frequent pattern. Ulcerative colitis showed a relatively higher proportion of 4P findings, whereas 5P findings were uncommon overall and absent in ulcerative colitis.

The overall distribution of MP extent differed significantly among Crohn’s disease, recurrent sigmoid diverticulitis, and ulcerative colitis (Monte Carlo exact test: *p* = 0.030). This result was consistent with Pearson’s chi-square test (χ^2^(4) = 10.53, *p* = 0.032). The corresponding effect size was small (Cramér’s V = 0.155). However, Holm-adjusted pairwise post hoc comparisons showed no statistically significant differences between Crohn’s disease and recurrent sigmoid diverticulitis (adjusted *p* = 0.078), Crohn’s disease and ulcerative colitis (adjusted *p* = 0.168), or recurrent sigmoid diverticulitis and ulcerative colitis (adjusted *p* = 0.078).

The renewed pseudonymized reassessment did not result in a relevant change in MP classification or in the overall prevalence estimate. Interim CT examinations performed as part of routine clinical care were available only in a subset of patients and demonstrated mesenteric changes compatible with MP in individual cases. Because CT acquisition was not systematic and was not performed at predefined study time points, no quantitative CT-MRI concordance analysis or temporal progression analysis was undertaken.

### 3.2. Topographical Distribution

**Figure 6:**
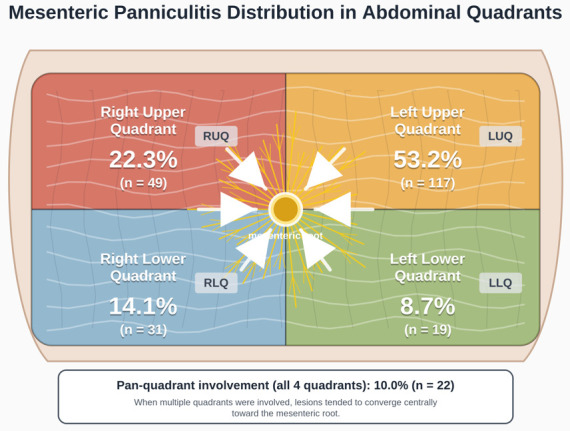
Schematic illustration of the localization and distribution of mesenteric panniculitis in the abdominal quadrants. Percentages for lower quadrants represent aggregated involvement based on multiquadrant distributions.

The localization of mesenteric panniculitis was classified according to the abdominal quadrants (Figure 6). Analysis of MRI scans from 220 patients demonstrated a clear predominance of lesions in the left upper quadrant (LUQ). In 53.2% of cases (n = 117), the LUQ was exclusively involved, whereas the right upper quadrant (RUQ) was the sole site of involvement in 22.3% of cases (n = 49). Involvement of all four abdominal quadrants was observed in 10.0% of patients (n = 22). Less common distributions included combinations of multiple quadrants, most notably RUQ + LUQ + RLQ in 8.2% of cases (n = 18) and RUQ + RLQ in 5.5% of cases (n = 12). Two additional quadrant combinations were each observed in a single patient (0.5% each). When multiple quadrants were involved, the affected areas tended to converge centrally toward the mesenteric root. Taken together, these findings demonstrate a marked predominance of mesenteric panniculitis in the left upper quadrant.

### 3.3. Clinical Characteristics of Patients with Mesenteric Panniculitis

Clinical characteristics varied across diagnostic groups. Diabetes mellitus differed significantly between groups and was observed exclusively in patients with sigmoid diverticulitis (49/88, 55.7%; *p* < 0.001). In contrast, smoking status did not differ significantly between groups (*p* = 0.054). The highest mean BMI was found in patients with sigmoid diverticulitis (27.9 kg/m^2^), compared with Crohn’s disease (23.7 kg/m^2^) and ulcerative colitis (24.1 kg/m^2^); however, no formal statistical comparison was performed because only mean values and ranges were available (Table 3).

## 4. Discussion

The present study shows that MRI findings consistent with mesenteric panniculitis (MP) are highly prevalent in patients with chronic inflammatory bowel disease and related chronic inflammatory conditions undergoing standardized 3-T MRI Sellink examinations. In contrast, such findings were rare in the control group. Taken together, these observations support the interpretation that MP may represent a secondary mesenteric inflammatory response rather than merely an incidental finding in this specific patient population. Clinically, MP may be associated with nonspecific abdominal symptoms or may occur in parallel with symptoms attributable to the underlying gastrointestinal disease [34,35].

A key finding of this study is the markedly higher prevalence of MRI-defined MP (87.3%) compared with previously reported rates of approximately 1–3% in predominantly CT-based cohorts [6,8,13,22,23,24,25,26]. This discrepancy should be interpreted with caution. First, the present cohort represents a selected population consisting exclusively of patients with chronic inflammatory bowel disease or chronic inflammatory bowel conditions rather than an unselected imaging population; more generally, MP has been described in association with a broad spectrum of inflammatory, autoimmune, postoperative, and neoplastic conditions [3,4,7,8,9,10,11,12,13,14,15,16]. Second, the high soft-tissue contrast of 3-T MRI may facilitate detection of subtle mesenteric inflammatory abnormalities that may be less conspicuous on CT, and characteristic MRI features of MP have been described as largely analogous to CT findings [7,31,34]. Third, the application of predefined composite MRI criteria may increase sensitivity for imaging-based detection compared with less standardized assessment approaches [10,34]. Importantly, increased mesenteric signal intensity on fat-saturated T2 sequences and displacement of bowel loops are nonspecific inflammatory or mass-effect signs and may contribute to overestimation if interpreted outside the full composite pattern. Therefore, the present results should be understood as the prevalence of MRI-defined features consistent with MP in a selected chronic inflammatory cohort, not as a histologically confirmed population prevalence of MP. Recent case series remain consistent with the broad clinical and radiological heterogeneity of MP, but they also show that contemporary cohorts are still small and therefore cannot resolve prevalence estimates in selected inflammatory populations [17,18]. Accordingly, the prevalence observed in the present study should not be interpreted as a general population prevalence of MP and is not directly comparable to CT-based epidemiologic estimates [6,8,13,22,23,24,25,26].

Although a statistically significant difference in the extent of MP was observed among Crohn’s disease, ulcerative colitis, and recurrent sigmoid diverticulitis, the corresponding effect size was small (Cramér’s V = 0.155) and adjusted pairwise comparisons were not statistically significant. This indicates that the observed group differences should be interpreted with caution and are unlikely to be of major clinical relevance. Accordingly, the present data do not support the conclusion that one specific disease entity is associated with a distinctly more pronounced MP pattern.

The findings further suggest that chronic inflammation itself may contribute substantially to the mesenteric inflammatory response, irrespective of its exact anatomical location. Similar associations have also been described in other inflammatory conditions, such as chronic cholecystitis [10], as well as in extraperitoneal processes, including non-neoplastic disorders and urolithiasis [36,37]. At the same time, this interpretation remains hypothesis-generating and requires confirmation in prospective studies.

The inclusion of patients with recurrent sigmoid diverticulitis also requires specific consideration. In this subgroup, MRI examinations reflected follow-up in symptomatic recurrence or a prolonged disease course rather than initial diagnostic imaging of first-time acute uncomplicated diverticulitis, for which CT is commonly used in clinical practice. The observed coexistence of recurrent diverticular inflammation and MP-compatible mesenteric changes is biologically plausible, but the retrospective design does not allow causal inference.

Differences in clinical characteristics between diagnostic groups may provide additional insight into the pathophysiology of MP. Patients with recurrent sigmoid diverticulitis showed a significantly higher prevalence of diabetes mellitus and descriptively higher BMI values than patients with Crohn’s disease or ulcerative colitis. Previous studies have reported associations between MP, metabolic syndrome, and increased visceral as well as subcutaneous adipose tissue [36,38]. Against this background, it appears plausible that systemic low-grade inflammation related to metabolic dysfunction may contribute to the development or extent of MP. However, because the control group was not formally matched for age, sex, or BMI, residual selection bias cannot be excluded.

The topographical distribution of MP in the present study showed a clear predominance in the left upper quadrant, which is in line with previous reports [39]. This pattern may reflect anatomical or vascular characteristics of the mesenteric root, although the underlying mechanisms remain incompletely understood.

MRI appears well suited for structured assessment of the extent, localization, and imaging phenotype of MP, particularly because of its high soft-tissue contrast and its ability to depict characteristic features such as mesenteric signal abnormalities, the hypointense pseudocapsule, embedded nodules, and displacement of adjacent bowel loops [10,34]. In this context, MRI may complement the predominantly CT-based literature on MP by enabling a more detailed soft-tissue characterization.

From a clinical perspective, it remains uncertain whether MP represents a clinically relevant pathological entity or predominantly an epiphenomenon of chronic inflammation. In many patients, MP appears to be asymptomatic and is detected incidentally [35,40], which raises the question of whether its identification should influence clinical management in a meaningful way. Recent reports likewise demonstrate that MP can present with atypical abdominal pain and can radiologically mimic malignant disease, including FDG PET/CT-positive lesions in an oncologic immunotherapy context [19,20,21]. Further studies are needed to clarify its prognostic significance and possible therapeutic implications.

### Limitations

Several limitations of this study should be acknowledged. First, the retrospective single-center design may limit generalizability. Second, the control group was relatively small and was not formally matched to the disease cohort for age, sex, or BMI; therefore, selection bias related to age distribution, body habitus, or clinical referral patterns cannot be excluded. Third, diagnostic classification was based on clinical and imaging criteria without systematic histopathological confirmation. Fourth, image analysis was performed by a single radiologist, and inter-reader variability was not assessed, despite the partly subjective nature of MP diagnosis. Although a renewed pseudonymized reassessment did not materially change the classification, this does not replace formal independent multi-reader analysis. Fifth, systematic CT correlation was not part of the predefined study design. Interim CT examinations were available in a subset of patients and showed MP-compatible changes in individual cases, but no quantitative CT-MRI concordance analysis was performed. Sixth, serial MRI follow-up was not available in a standardized manner, so the study cannot determine whether the observed findings represent stable mesenteric panniculitis or transient inflammation-related mesenteric changes. Finally, the inclusion of recurrent sigmoid diverticulitis broadens the cohort beyond classical inflammatory bowel disease and should therefore be taken into account when interpreting disease-specific conclusions. In addition, the criteria-based MRI definition used in this study may have increased sensitivity for subtle mesenteric inflammatory changes because increased T2 signal intensity and bowel-loop displacement are nonspecific features; this may limit direct comparability with prior CT-based prevalence studies and with more restrictive definitions based only on pseudocapsule, embedded nodules, and the fat ring sign.

## 5. Conclusions

MRI-defined features consistent with mesenteric panniculitis are frequent in patients with chronic intestinal inflammatory conditions undergoing standardized 3-T MRI Sellink examinations when predefined composite imaging criteria are applied. In this selected cohort, 220 of 252 patients (87.3%) fulfilled the MRI criteria for MP, whereas such findings were rare in controls. Although the overall distribution of MP extent differed significantly among diagnostic groups, no adjusted pairwise differences were observed. Overall, the findings suggest that MP-like mesenteric changes may represent a common imaging correlate of chronic intestinal inflammation. MRI enables structured detection and topographic assessment of MP and may complement CT-based characterization, but prospective studies with matched controls, multi-reader assessment, systematic CT correlation, and longitudinal follow-up are needed before clinical implications can be defined.

## Figures and Tables

**Table 1:** Age of patients in the investigated diagnostic groups.

Diagnosis	*n*	Mean Age and Range (Years)
Crohn’s disease	110	28.1 (18–38)
Sigmoid diverticulitis	99	64.8 (38–77)
Ulcerative colitis	43	38.8 (20–58)

**Table 2:** Descriptive characteristics of the control group.

Parameter	Value
*n*	60
Age, mean (range) years	37.4 (18–45)
Gender distribution	42 women, 18 men
BMI, mean (range) kg/m^2^	24.1 (17.1–28.4)

**Table 3:** Clinical characteristics of patients with mesenteric panniculitis according to diagnostic group.

Characteristic	Crohn’s Disease	Sigmoid Diverticulitis	Ulcerative Colitis	*p*-Value
Diabetes mellitus	0/104 (0%)	49/88 (55.7%)	0/28 (0%)	<0.001
BMI, mean (range), kg/m^2^	23.7 (18.1–34.1)	27.9 (18.6–39.9)	24.1 (21.9–31.2)	n.a.
Smoking	40/104 (38.5%)	38/88 (43.2%)	5/28 (17.9%)	0.054

## Data Availability

The data presented in this study are available upon request from the corresponding author due to patient privacy concerns and ethical restrictions.

## Abbreviations

BMI	Body mass index
CRP	C-reactive protein
CT	Computed tomography
CU	Ulcerative colitis
DWI	Diffusion-weighted imaging
FFE	Fast field echo
Gd-DTPA	Gadolinium-diethylenetriaminepentaacetic acid
IBD	Inflammatory bowel disease
LLQ	Left lower quadrant
LUQ	Left upper quadrant
MRE	Magnetic resonance enterography
MRI	Magnetic resonance imaging
MP	Mesenteric panniculitis
*n*	Number
PACS	Picture archiving and communication system
RIS	Radiology information system
RLQ	Right lower quadrant
RUQ	Right upper quadrant
SPAIR	Spectral attenuated inversion recovery
T2-TSE	T2-weighted turbo spin-echo
T2-SPAIR	T2-weighted SPAIR
THRIVE	T1 high-resolution isotropic volume examination
